# Mobility-Based Ionization Detector with a Soft X‑ray Source

**DOI:** 10.1021/acs.analchem.6c00285

**Published:** 2026-04-14

**Authors:** Krzysztof Piwowarski, Jarosław Ławreńczyk, Michał Kędzierski, Jarosław Puton

**Affiliations:** 1 Faculty of Advanced Technologies and Chemistry, Military University of Technology, ul. gen. Sylwestra Kaliskiego 2, 00-908 Warsaw, Poland; 2 Faculty of Civil Engineering and Geodesy, Military University of Technology, ul. gen. Sylwestra Kaliskiego 2, 00-908 Warsaw, Poland

## Abstract

This work concerns an analytical instrument intended for the detection of chemical substances characterized by the ability to form stable ions with mobilities lower than those generated in clean air. The operation of the detector is based on the ionization of gas and the measurement of the ionic current flowing at alternating electric field. The detector’s electrode geometry is cylindrical, with the ion collector incorporated into the external electrode. An integral part of the detector is an ionization source emitting low-energy X-ray radiation. The effect of adding two test substances (2‑heptanone and methyl salicylate) to air on ionic current values was studied in various detector operating modes. With a constant voltage power supply, the changes in current induced by the introduction of the test substance were small. Significantly higher analytical signal values were obtained when the detector was powered by a square-wave alternating voltage. The ionic current waveform under these conditions provides information about the presence of substances in the air with high proton affinity, which form stable positive ions, as well as the presence of electrophilic substances. It is also possible to estimate the minimum mobilities of the ions formed by the sample molecules. Particularly favorable detection conditions are obtained in the operating mode in which the ionization source is periodically switched on. In this mode, the analytical signal values are significantly higher than in other operating modes. A theoretical analysis of the detector’s operation was conducted, based on ion balance equations.

## Graphical abstract

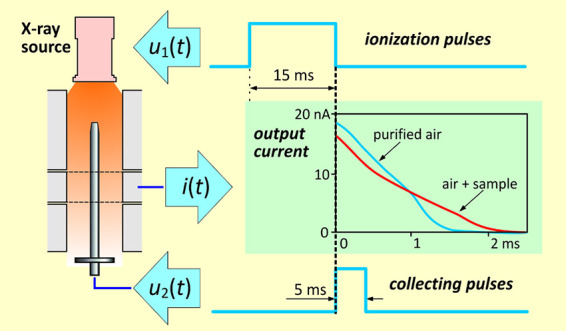

## Introduction

An ionization detector can be considered as a system of electrodes with a gas enclosed between them. The detector also includes an internal ionization source, which generates positive and negative ions. The current resulting from the movement of these ions in an electric field constitutes the useful signal of the detector. Its value is determined by the ionization intensity, the electric field distribution, and the properties of the charge carriers. The development of practical applications of ionization methods in analytics began in the mid-20th century. In 1958, the design of the flame ionization detector (FID) was presented,^1^ whose operation is based on the formation of ions in complex processes occurring in a flame.^2^ Two years later, a photoionization detector (PID), using ultraviolet radiation to ionize organic components of the gas sample was described,^3^ as well as an electron capture detector (ECD),^4^ known as a very effective device for detecting compounds with high electron affinity. The three types of detectors mentioned above are successfully used in analytics to this day, and the fact that they were developed almost simultaneously stem from the needs of gas chromatography, which was then developing very rapidly. Descriptions of the design, operating principles, and measurement characteristics of simple ionization detectors for chromatographic applications can be found in Poole’s work.^5^ Simple ionization detectors meet all the criteria to be defined as chemical sensors. Their design is simple, and the operating principle is based on the conversion of chemical information into a useful analytical signal. According to the most commonly used classifications of chemical sensors,^6–8^ their main groups include electrochemical, optical, thermal, gravimetric, and conductometric sensors. It seems that ionization detectors can be treated as another group of chemical sensors.

The development of ionization methods in analytics is related, among other things, to the search for effective ways to detect hazardous materials. This is due to the fact that substances such as explosives and chemical warfare agents (CWAs) form, as a result of chemical ionization, relatively heavy ions with characteristic mobilities. Devices utilizing this property of hazardous materials are ion mobility spectrometers (IMS detectors), which have been the standard equipment of chemical troops and security services for many years.^9,10^ The design of the IMS detector with a drift tube, most commonly used in portable explosives and CWA analyzers, is relatively complex. In contrast, the aspiration IMS detector is a compact device consisting of a small number of parts.^11^ Devices used for CWA detection in the armed forces of some countries also have a simple design.^12,13^ The possibility of using such an ionization sensor to detect organophosphorus CWAs has been reported by Milinković and Milanko.^14^ Their detector is a cylindrical ionization chamber with an internal radioactive source (241-Am) emitting alpha particles. Ions inside the detector move in a periodically varying electric field whose period is comparable to the ions time-of-flight between electrodes. The output signal is the average current measured in the circuit connected with central electrode. A schematic of the detector design and the dependence of the average current on the frequency of the supply voltage are shown in Figure 1. When only clean air is present in the detector, the mobilities of positive and negative ions are approximately equal and the value of the average current is small. If the air inside the detector contains an admixture of chemical compounds with high proton affinity (e.g., organophosphorus CWAs), stable, heavy positive ions with mobilities significantly lower than those observed for ions found in pure air. The presence of these compounds causes a change in the average value of detector current and triggers an alarm signal.

**Figure 1:**
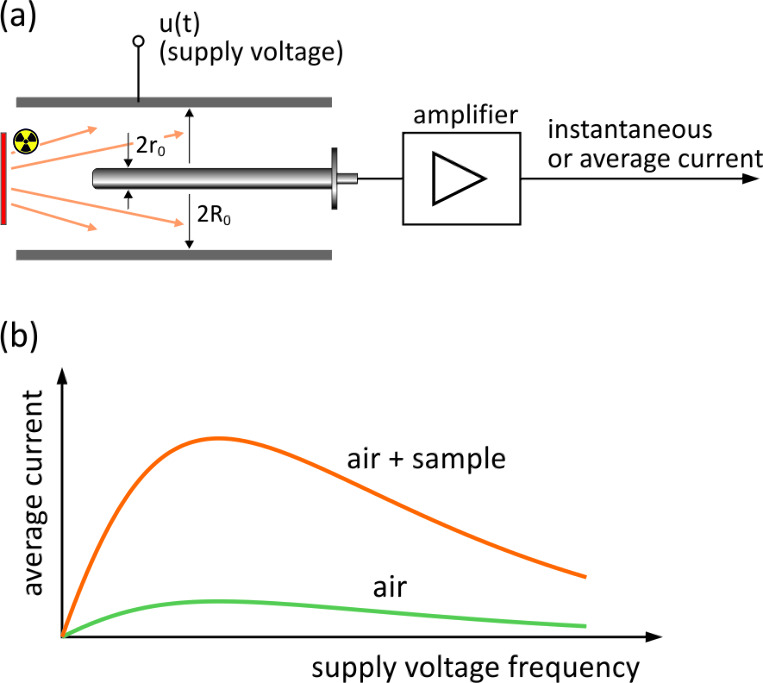
Schematic of a simple ionization detector with a radioactive source powered by an alternating voltage (a) and the dependence of the average current on frequency for different gas compositions inside the detector (b).

Theoretical description of the ionization detector operation is based on the analysis of the ions motion in the gas filling the detector. The detector output signal is a current measured in the external circuit. In theoretical considerations, the value of this current can be determined from the conservation of energy principle, according to which the work done by the electric field on the charge carriers must be equal to the energy taken from the external circuit.^15^ For a single positive charge moving in the *x*-direction, the conservation of energy can be expressed by the equation:

1 E(x,t)e⁡dx=i(t)u(t)⁡dt

where *E* is the electric field intensity, *e* is the elementary charge (*e* = 1.6·10^–19^ C), *dx* is the elementary displacement, *i*(*t*) is the current flowing in the external circuit, *u*(*t*) is the voltage between the detector electrodes, and *dt* is the elementary time increment. The velocity of ion motion in the gas *v* is determined by the electric field intensity *E* and the ion mobility *K*:

2 $$v=\frac{dx}{dt}=KE$$

In a cylindrical detector, such as in Figure 1, the electric field has only a radial component. If the detector contains positive and negative ions of many types, with concentrations *n*_*i*_ that are functions of radius and time, then the instantaneous detector current can be described by the formula:

3 $$i\left(t\right)=\frac{2\pi eh}{u\left(t\right)}\int_{r_{0}}^{R_{0}}rE^{2}\left(r,t\right)\underset{i}{\sum }\left(K_{i}n_{i}\left(r,t\right)\right)dr$$

where *r*_0_ and *R*_0_ are the radii of the central and outer electrodes of the detector, *h* is the length of the ionization region, and *u*(*t*) is the time-dependent voltage between its electrodes. The analysis of the cylindrical ionization detector presented by Milinković^16^ concerns the case of sinusoidal voltage *u*(*t*). The most important simplification in Milinković’s model is the assumption that calculations can be performed for average ion concentrations without the need to determine their time-dependent spatial distributions.

The main disadvantage of the technical solutions described above is the use of radioactive sources, which requires obtaining formal permits from institutions responsible for nuclear safety. To avoid this inconvenience, a source of soft X-ray was used to ionize the gas in the detector that is the subject of this research. This type of ionization method is increasingly used in analytical devices. Soft X-ray sources can be found in both simple detectors, such as ECDs,^17^ and in IMS detectors.^18^

Besides the need to modify the detector design, the inspiration for research on the detector came from the desire to understand the phenomena occurring within it. During the research, various variants of the detector’s operation were tested. At the same time, theoretical work and calculations were carried out, the results of which allow for the justification of the dependencies obtained experimentally. The theoretical part of our work is presented prior to the summary of the experimental results.

## Experimental Section

The view of the detector under study is shown in Figure 2a, and its axial cross-section in Figure 2b. The detector consists of three electrodes. The central electrode is used for supply and is connected to an appropriate source of constant or alternating voltage. The housing, which is a hollow metal cylinder, is always grounded. A collecting electrode with the same internal diameter as the housing is located in the center of it. The potential of the collecting electrode is always zero because it is connected to the input of grounded transimpedance amplifier. This design provides an almost perfectly cylindrical geometry of the electrode system. The ion current, which constitutes the output signal of the detector, is collected exclusively within the space bounded by the collecting electrode ring. Gas ionization is caused by the soft X-ray source placed axially opposite the central electrode. In the charge collection region, the radiation field is nonuniform, mainly due to partial shading of the source by the central electrode. The dimensions and characteristic operating parameters of the detector are summarized in Table 1.

**Figure 2:**
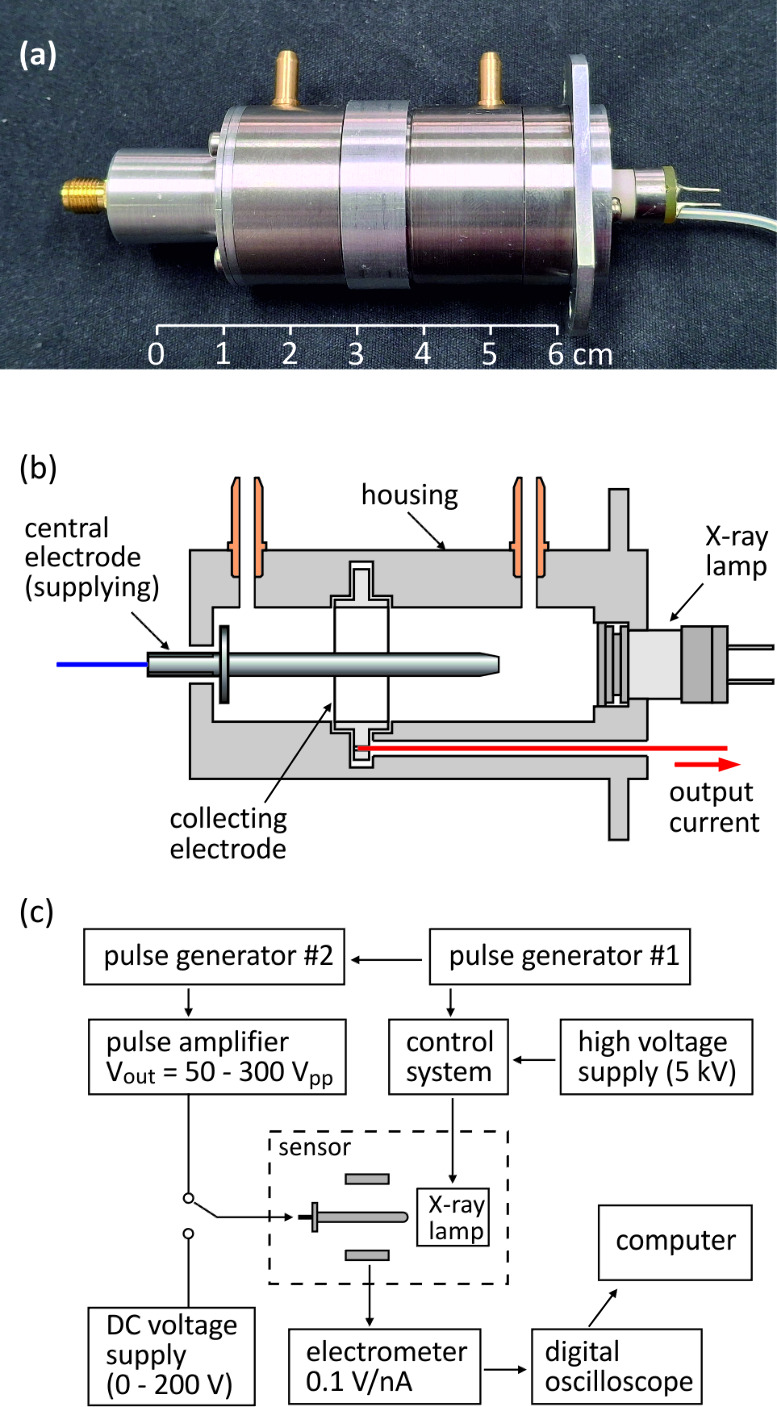
View of the detector (a) and its axial cross-section (b). A diagram of the measurement system for testing the detector characteristics (c).

**Table 1:** Technical Parameters of the Detector under Test

Parameter	Value
detector dimensions	
length	10.5 cm
diameter	3.5 cm
ionization chamber length	4.9 cm
ionization chamber diameter	1.5 cm
ionization chamber volume	6.9 cm^3^
inner electrode diameter	0.30 cm
active volume^a^	1.2 cm^3^
soft X-ray source	
type	SCXT 0817 (Sunje)
accelerating voltage (HV)	4.97 kV
emission current	0.7 mA
cathode filament current	500 mA
switching on and off method	changes in HV
detector voltage and current	
DC voltage	0–150 V_DC_ ^b^
symmetrical square wave	10–2000 Hz, 100 – 300 V_pp_
unipolar square pulses	5–10 ms, 50 – 150 V^b^
saturation current	3.01 nA
Operation temperature	ambient
Gas flow	200 mL/min

a The volume from which ions are collected for current measurements.

b Positive or negative polarity.

A block diagram of the electronic circuits interacting with the detector is shown in Figure 2c. The detector is powered by connecting its central electrode to a DC or variable voltage source. DC voltage was generated using a Model 248 high-voltage power supply (Keithley). The DC power supply was used only to measure the static current–voltage characteristics of the detector. Most measurements were conducted using variable voltage supply obtained from a homemade pulse amplifier which was controlled by a RS DG805 function generator (pulse generator #2). The output voltage of the pulse amplifier was either a symmetrical square wave or a series of positive or negative pulses with adjustable width. The high voltage required to power the soft X-ray source was obtained from an SL8PN30/ESL/23 (Spellman) power supply with a control circuit of our own design. This system enabled both continuous operation of the source and fast switching on and off of the radiation generated by the X-ray tube. The turn-on time of the source was determined by the pulse length from pulse generator #1 (RS DG805). Both generators were connected to synchronize the soft X-ray source turn-on pulses with the detector supply voltage pulses. The detector’s output signal, i.e., the ion current, was directed to a homemade transimpedance amplifier and recorded using an XDS3062 (OWON) digital oscilloscope. The amplifier gain was -0.1 V/nA (inverting amplifier) and the graphs later in the article show the results obtained directly from the amplifier output, i.e. the current shown in the graphs has reverse polarity to the convention adopted in electrical circuit theory. In instantaneous current measurements, the time constant was approximately 10 μs, however in the frequency characteristics studies, the average current was measured and therefore the time constant was increased to approximately 0.4 s. Static measurements of the detector current–voltage characteristics were performed using a Model 6487 picoammeter (Keithley). The carrier gas used in the studies was air-dried through molecular sieves. The test substances used in the studies were 2-heptanone (99%, Sigma-Aldrich) and methyl salicylate (99%, Carl ROTH).

## Theory

The calculation of the output signal of the detector was based on the theoretical analysis of ion movement in the space between the electrodes. The starting point of these considerations were the balance equations resulting from the continuity law. The general form of the balance equation for the *i*-th ion type can be written as

4 $$\frac{\partial n_{i}}{\partial t}=-\mathrm{div}\left(n_{i}v_{i}\right)+\mathrm{div}\left(D_{i}\mathrm{grad}\left(n_{i}\right)\right)+P_{i}-L_{i}$$

where *D*_*i*_ is the diffusion coefficient of the *i*-th ion species, *P*_*i*_ is the source term describing the production of ions as a result of ionization and/or ion-molecular reactions, and *L*_*i*_ is the component of losses resulting from recombination and reaction processes. In the general case, the ion velocity *v*_*i*_ is the sum of the flowing gas velocity and the drift velocity of the ions in the electric field.

The simplicity of eq 4 is deceptive. In reality, the problem is described by a system of equations, whose number is equal to the number of ionic and neutral species involved in ion-molecular reactions. These equations are coupled due to the presence of source and sink terms. It is often necessary to take into account the so-called space charge effect, i.e. the fact that the electric field is generated not only by the electrode system, but also by the spatial distribution of charge carriers. Furthermore, geometry of real detectors is rarely ideal, and the available information on parameters such as diffusion coefficients, recombination coefficients, and reaction rate constants is incomplete. All this makes obtaining accurate solutions to the ion balance problem difficult. Therefore, it is necessary to use approximations and simplifications that allow for the explanation of certain phenomena observed in measurements. The primary goal of the following discussion is to obtain estimates of ion concentration distributions, which are necessary for calculating the current based on eq 3.

A schematic illustrating the geometry of the detector under study is shown in Figure 3a. It was assumed that the mathematical description of the phenomena occurring in the detector can be based on a one-dimensional model, in which the electric field, ionization intensity, and ion concentration distributions depend only on the radius. In our experiments, we used sufficiently high supply voltage amplitudes to produce high electric field intensity within the detector. Under such conditions, ion concentrations are low and their flight times between the electrodes are short. This allows recombination losses, ion diffusion, and space charge effects to be neglected in modeling the phenomena occurring within the detector. Furthermore, it was assumed that the process of stable ion formation is rapid, and therefore the change in the composition of the ion swarms during drift can be neglected. This latter assumption is well satisfied in the absence of a sample, when only reactant ions are present, or at a relatively high sample concentration, when ion-molecular reactions leading to the formation of sample ions occur very rapidly. Under these assumptions, the ion balance equation reduces to a simple one-dimensional advection equation with a source term:

5 $$\frac{\partial n_{i}\left(r,t\right)}{\partial t}=S\left(r\right)-\frac{B_{i}u\left(t\right)}{2r}\frac{\partial n_{i}\left(r,t\right)}{\partial r}$$

where *n*_*i*_(*r,t*) is the concentration of *i*-th type of ions, *S*(*r*) is the ionization density caused by the X-ray source, *u*(*t*) is the supply voltage applied to the central electrode, and *B*_*i*_ is a factor that accounts for the detector geometry and ion properties:

6 $$B_{i}=\frac{\pm 2K_{i}}{\ln\left(R_{0}/r_{0}\right)}$$

**Figure 3:**
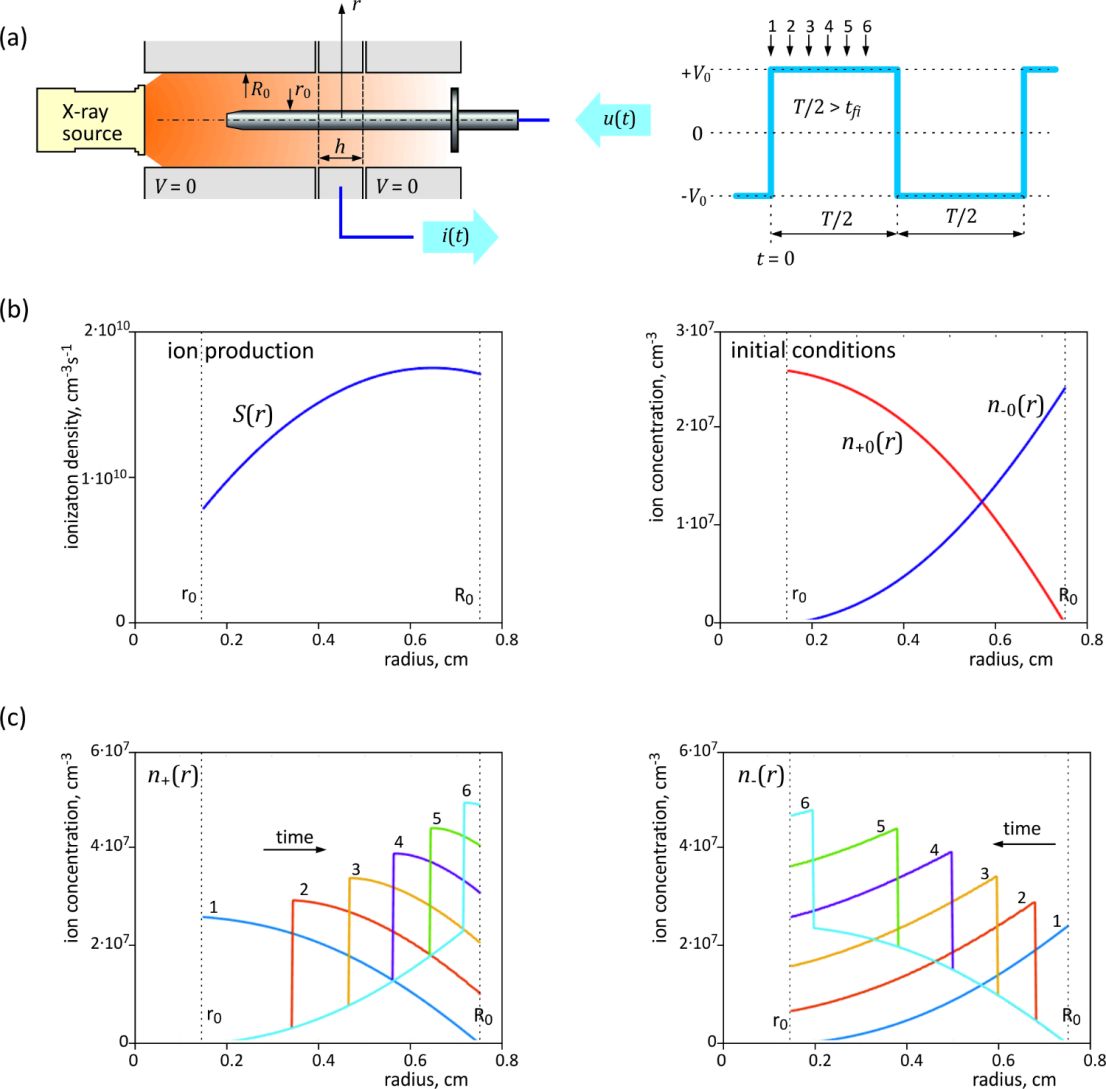
Theoretical description of the detector: (a) geometry of the electrode system and time course of the supply voltage; (b) spatial distribution of ionization density and the initial condition, i.e., the spatial distribution of ion concentrations at time *t* = 0; and (c) distributions of positive and negative ion concentration at successive time intervals after the voltage is switched. The numbers above the curves in (c) correspond to the times marked in (a) on the right side.

The sign of the *B*_*i*_ coefficient corresponds to the polarity of ions (″+″ for positive ions, ″-″ for negative ions). The solution of eq 5 depends on the shape of the supply voltage *u*(*t*). The calculations, the results of which are presented below, refer to a supply voltage in the form of a symmetrical square wave shown in Figure 3a, where the half-period of the voltage waveform is longer than the time-of-flight of ions between the detector electrodes:

7 $$\frac{T}{2}>t_{fi}=\left|\frac{R_{0}^{2}-r_{0}^{2}}{B_{i}V_{0}}\right|$$

where *V*_0_ is the potential of the central electrode.

The ionization density distribution *S*(*r*) was estimated based on geometric considerations, assuming that the radiation emission from the source surface is uniform. In these calculations, both the distance from the source and its shading by the central electrode were taken into account. A more detailed explanation of how the ionization density distribution was estimated is provided in the Supporting Information (S1). The ionization density was then normalized to match the experimentally determined saturation current of the detector:

8 $$I_{sat}=2\pi he\int_{r_{0}}^{R_{0}}S\left(r\right)rdr=3.01\mathrm{nA}$$

Using the ionization density *S*(*r*), initial conditions *n*_+0_(*r*) and *n*_–0_(*r*) were determined for the positive and negative ion concentrations. They correspond to stationary concentration distributions calculated by solving eq 5 with zero value of the derivative of concentration with respect to time. The calculations were performed for a central electrode potential of −125 V and typical ion mobilities in air at 298 K (*K*_+_ = 2.10 cm^2^V^–1^s^–1^, *K*_–_ = 2.25 cm^2^V^–1^s^–1^). Graphs of ionization density and initial concentration distributions are shown in Figure 3b.

The solution of the advection eq 5 for a given initial condition *n*_i0_(*r*) and a known ionization density distribution *S*(*r*) can be found by considering the charge conservation in a cylindrical layer moving in accordance with the motion of the ions. The derived formula for calculating the time-dependent ion concentration distributions is

9 $$n_{i}\left(r,t\right)=n_{i0}\left(r^{\prime }\left(t\right)\right)+\int_{0}^{t}S\left(r^{\prime }\left(\tau \right)\right)d\tau$$

where $r^{\prime }\left(t\right)=\sqrt{r^{2}-B_{i}V_{0}t}$ . The derivation of formula [Disp-formula eq9] is provided in the Supporting Information (S2). Numerical calculations based on formula [Disp-formula eq9] were performed using the *AdvResults* program (function) written using the basic MATLAB software package. For the assumed supply voltage waveform, i.e., when the central electrode potential changes from negative to positive at time t = 0, the positive ion cloud moves toward the outer electrode, and negative ions move toward the central electrode. The spatial distributions of ion concentrations at successive time intervals are shown in Figure 3c. The *AdvResults* program is also used to calculate the time courses of the ion current using formula [Disp-formula eq3] and the value of the average current.

## Results and Discussion

### Static Current–Voltage Characteristics of the Detector

Measurements of the detector current dependence on voltage were performed for dried purified air without sample and air with the addition of 2-heptanone as an example test substance. The 2-heptanone concentration was approximately 29 ppb. The characteristic curves are shown in Figure 4. The shape of the obtained curves is typical for ionization chamber characteristics.^19^ In the range from – 50 to +50 V, a strong dependence of the ion current on the voltage is observed. This section of the characteristic curve is called the recombination range. For voltages with absolute values greater than 75 V, the characteristic reaches the saturation range, where current changes are small. The value of the saturation current (3.01 nA) was used to estimate the absolute value of the ionization density based on formula [Disp-formula eq8]. The shape of current–voltage characteristics can be explained by considering two competing processes: ion transport in an electric field and recombination.^19^

**Figure 4:**
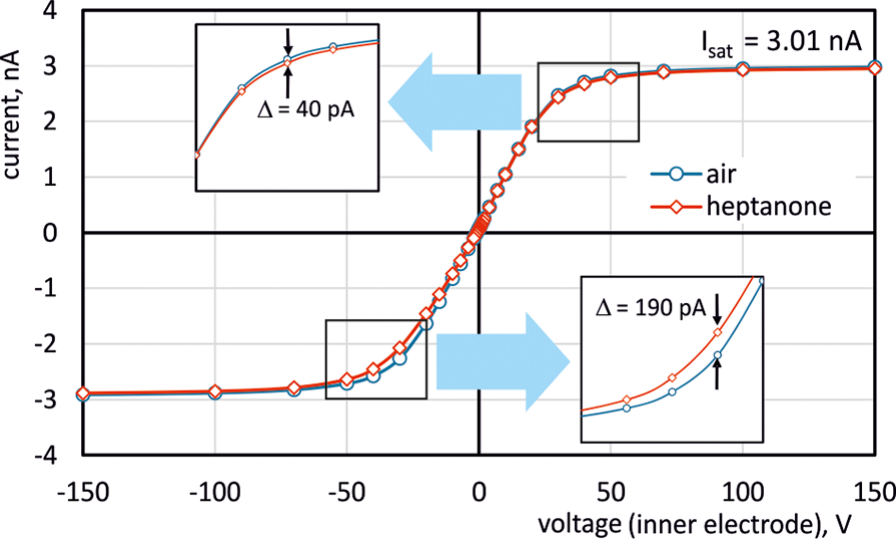
Current–voltage characteristics of the tested detector.

Introducing a sample (2-heptanone) into the air flowing through the detector causes a small but significant change in the current–voltage characteristic. In the presence of the sample, the absolute value of the ion current in the recombination region decreases. This is due to greater recombination losses when the positive charge carriers are ions formed from 2-heptanone molecules. Measurements conducted in our laboratory using ion mobility spectrometers^20^ have shown that at ketone concentrations higher than 10 ppb, the dominant positive ions are M_2_H^+^ dimers, consisting of two ketone molecules (M) bonded by a proton (H^+^). The mobility of these ions is significantly lower than that of hydrated hydronium ions, which are typical for clean air. Introducing the sample causes the positive ion carriers to remain in the detector volume for a longer time, where the concentration of negative ions is relatively high. This creates favorable conditions for recombination. It is easy to see that the effect of sample addition on the ionic current is greater when the detector’s central electrode is negatively polarized. This phenomenon is of a general nature and it can be stated that the value of the ionic current is dominated by ions moving toward the central electrode. As shown in Figure 4, the maximum change in current caused by sample introduction is 190 pA, or approximately 6% of the saturation current. It seems that such a small change in current cannot be used as a useful analytical signal enabling effective detection.

### Ion Current Waveform when Supplied with Alternating Voltage

Most of the experimental studies were performed with the detector supplied with alternating voltage in the form of a symmetrical square wave. Exemplary results of such studies are shown in Figure 5a. The supply voltage amplitude was 125 V, and the square wave period was 7 ms. Two ranges can be distinguished in the current waveform. Immediately after the voltage polarity change, an ionic current pulse is observed. It is evident that the shape and duration of this pulse depend on the gas composition and the direction of the voltage change. This pulse is related to a change in the distribution of ion concentrations inside the detector. After the pulse ends, the current stabilizes and, at sufficiently high voltage amplitudes, reaches a value equal to the saturation current measured statically. The pulse durations after the polarity change of the detector’s central electrode from positive to negative are approximately 1.65 ms for clean air and 2.65 ms for air with 2-heptanone added. The time for the ion swarm reformation is equal to the time-of-flight of ions between the detector electrodes, which can be calculated using Equations 6 and ([Disp-formula eq7]). These formulas also enable estimation of mobility based on the time-of-flight measurement, i.e., the duration of the current pulse after the polarity change. For the pulse durations given above, the calculated mobility values are *K*_*+,ai*__r_ = 2.10 cm^2^V^–1^s^–1^ for air and *K*_*+,hept*_ = 1.32 cm^2^V^–1^s^–1^ for 2-heptanone. In our laboratory, using an ion mobility spectrometer with a drift tube, we obtained mobility values (at 30 °C and 10 ppm of H_2_O) equal to 2.20 cm^2^V^–1^s^–1^ for hydronium ions and 1.39 cm^2^V^–1^s^–1^ for heptanone dimer ions). It can therefore be concluded that the duration of the ionic current pulse is indeed related to the ion mobility.

**Figure 5:**
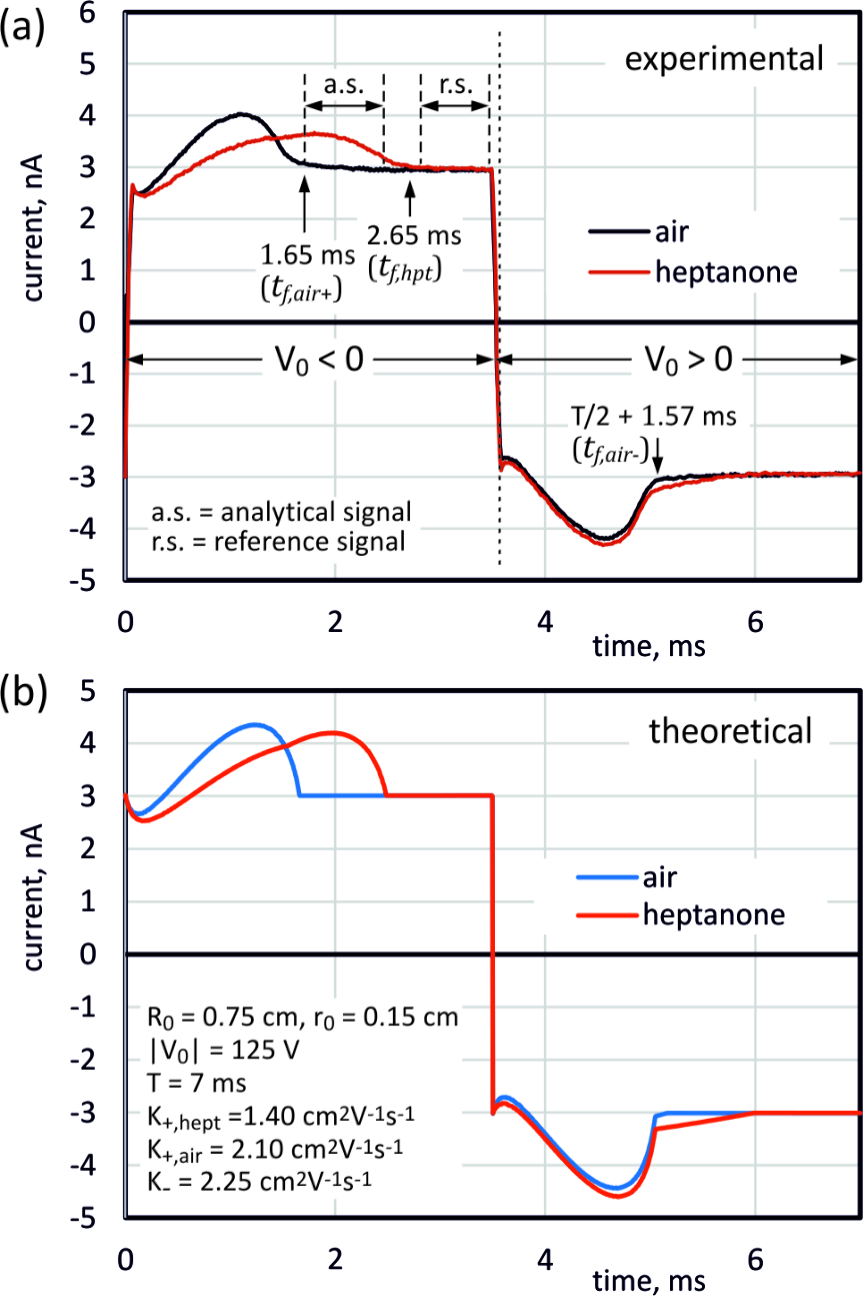
Measured (a) and theoretically determined (b) dependencies of the detector current on time.

The effect of the presence of 2-heptanone (analyte) on the current time course is very pronounced. At the time *t* = 1.9 ms, the difference between currents measured in the presence of the sample and in clean air is 0.7 nA. An average current determined over the interval 1.7–2.5 ms can therefore be used to detect the presence of chemical compounds that form stable, low-mobility positive ions. This current can be treated as an analytical signal (a.s. in Figure 5a). Measuring the average current for longer times can provide a reference signal value (r.s. in Figure 5a), which allows for monitoring the current level in the detector and calculating a relative signal which is almost independent of the saturation current level.

When assessing the usefulness of the tested detector in analytics, it is important to note a distinctive feature of the current time course. When the voltage polarity at the central electrode changes from positive to negative (*V*_0_ > 0 → *V*_0_ < 0), a clear effect of the analyte’s presence on the signal is observed. No such effect is observed for the reverse polarity change (*V*_0_ < 0 → *V*_0_ > 0). This is because, as in static measurements, the current value is determined by ions moving toward the central electrode. Heptanone, like other ketones, belongs to the group of substances that form stable positive ions. The mobility of negative ions in the presence of heptanone remains unchanged. The Supporting Information (S3) provides plots of the current time-courses measured after the introduction of methyl salicylate, whose molecules form both positive and negative stable ions, into the gas flowing through the detector. The effect of the detector supply voltage amplitude on the time courses of the ionic current is also shown in the Supporting Information (S4).

Figure 5b shows the current waveforms determined using the advection model. Using eq 9, time-dependent ion concentration distributions were calculated, and then the ion currents were obtained using formula [Disp-formula eq3]. It can be concluded that, despite the numerous simplifications adopted in the ion transport model, the calculated results are in very good agreement with the experimental data.

### Frequency Characteristic of the Detector

Measurements of frequency characteristics were performed with the time constant of the electrometric amplifier increased to 0.4 s. Example results of such measurements are shown in Figure 6a.

**Figure 6:**
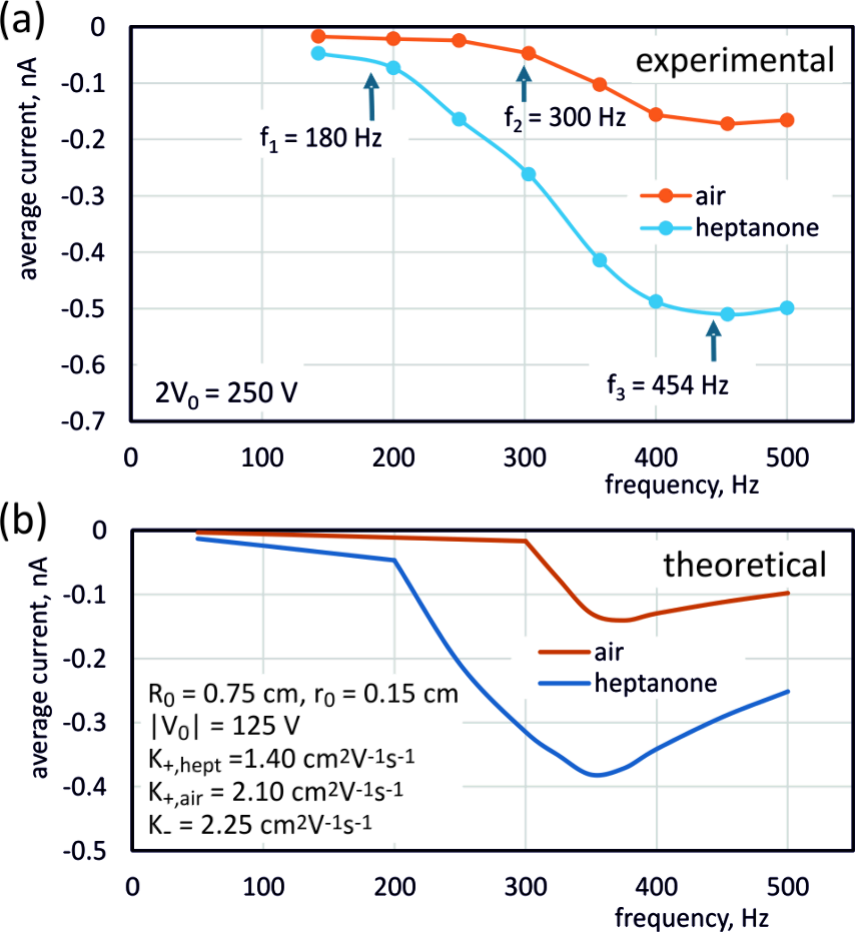
Measured (a) and theoretically determined (b) frequency characteristics of the detector.

In the low-frequency range (*f* < 150 Hz), the detector average current is small and increases only slowly with increasing frequency. Above a certain cutoff frequency, the increase in the mean current is much greater. This frequency is equal approximately 180 Hz when the sample is present in the gas flowing through the detector, and approximately 300 Hz for clean air. These frequencies correspond to half-period times of 2.77 and 1.67 ms, which are similar to the times of flight estimated from measurements of the ion current waveform (see Figure 5a). Therefore, a significant increase in the average current occurs at a frequency above which some ions do not reach the electrodes within half a period. This means that above the cutoff frequency, a swarm of oscillating ions appears inside the detector. The effect of the presence of oscillating ions on the instantaneous current waveform is shown and discussed in the Supporting Information (S5). The cutoff frequency observed in the characteristic is lower for lower value of ion mobility. At higher frequencies, the average current increases and reaches a maximum value at a frequency of approximately 450 Hz. This value cannot be explained by a simple consideration of ion mobility and transit time between the electrodes. For higher frequencies, the average current decreases. Analyzing of the sample effect on the frequency characteristic, it can be seen that the analytical signal can be the average current measured at the appropriate frequency. The maximum change in the average current caused by the presence of the sample was 0.34 nA and was obtained at frequency of 454 Hz.

Theoretical calculations of the frequency characteristics were performed using formulas [Disp-formula eq9] and ([Disp-formula eq3]), followed by averaging the instantaneous current value. It was assumed that oscillating ions do not contribute to the average current. Graphs of the characteristics calculated in this manner are presented in Figure 6b. Although the theoretical curves are similar in shape to the experimental ones, a significant discrepancy is observed in the position of the maximum of average current. In the theoretically calculated relationships, the maximum occurs at a frequency of 340 Hz, corresponding to a negative ion flight time of approximately 1.57 ms (see Figure 5a). For the experimental relationships, the maximum is observed at a frequency of 454 Hz. Therefore, simple theoretical model is not very accurate in this case. The probable cause of this discrepancy is the diffusional expansion of ion swarms oscillating within the detector.

### Measurements with Periodical Switching on of the Ionization Source

The use of a soft X-ray ionization source in the detector enabled measurements to be conducted in an operating mode unavailable for detectors with isotopic sources. Our studies utilized synchronous control of the soft X-ray source and the detector voltage supply. The measurement principle is shown in Figure 7a. Pulse generator #1 (see Figure 2c) controlled the high voltage supplying the soft X-ray source. The source was switched on with pulses of 3 to 15 ms duration and repetition period of 30 ms. The falling edge of these pulses turned on the voltage applied to the detector’s central electrode. The duration of the supply voltage pulse was 5 ms, which is longer than the transit time of ions between the detector electrodes. This ensured effective collection of ions present in the space between the electrodes when the ionization source was switched off. The time courses of the current measured in the collecting electrode circuit are shown in Figure 7b. As the duration of the ionization control pulse increases, the current pulse amplitude and the collected charge also increase. The maximum current in this operating mode is approximately 18 nA and is significantly higher than that obtained for a square-wave voltage supply with the ionization source turned on continuously (see Figure 5). The increase in the current pulse amplitude is not proportional to the ionization source’s on-time. At longer ionization times, saturation is observed, i.e., a slight increase in the pulse amplitude and charge with increasing ionization time. This phenomenon can be explained by the effect of reaching recombination equilibrium, whereby the production of ions as a result of ionization is equal to their losses, caused primarily by the recombination process. A brief description of the phenomenon of reaching recombination equilibrium is included in the Supporting Information (S6).

**Figure 7:**
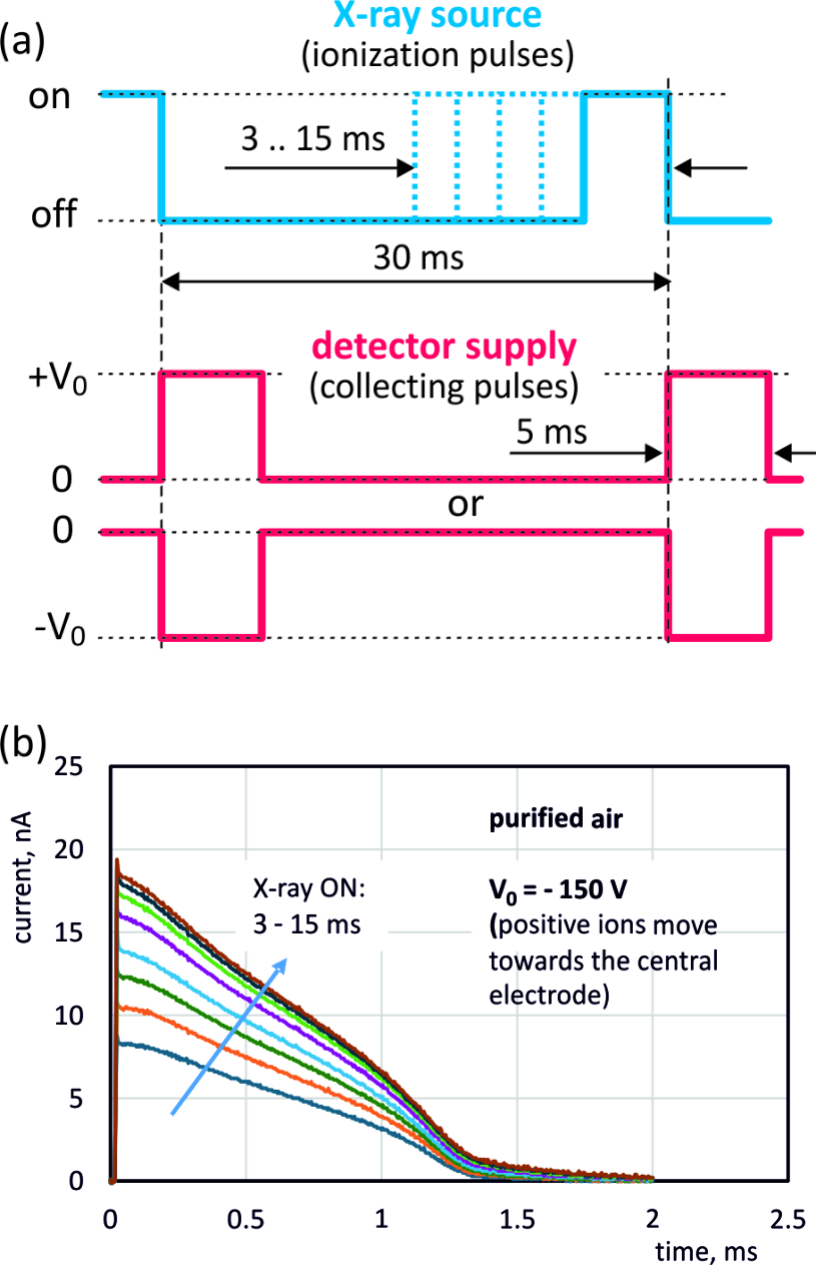
Measurements performed with periodic switching of the ionization source. Sequence of pulses controlling ionization and the voltage collecting the ions (a). Resulting current waveforms measured at the collecting electrode (b).

The shape of the detector current versus time strongly depends on the gas composition. The addition of a substance that form stable, low-mobility positive ions significantly reduces the amplitude and increases the duration of the current pulse measured with a negative voltage applied to the central electrode (Figure 8a). At 1.5 ms after switching on the voltage, the difference between the currents measured for air containing the 2-heptanone sample and for purified air is 3.0 nA. This value is about five times larger than the difference in instantaneous currents obtained with a square wave power supply and a continuously switched-on ionization source (see Figure 5). Therefore, it can be concluded that detecting substances with high proton affinity in this manner can be very effective. By contrast only minor changes in the amplitude and shape of the current pulse are observed when a positive voltage is applied to the central electrode (Figure 8b). The detector response to methyl salicylate, whose presence in the ionized gas results in the formation of stable positive and negative ions, is presented in Supporting Information (S7).

**Figure 8:**
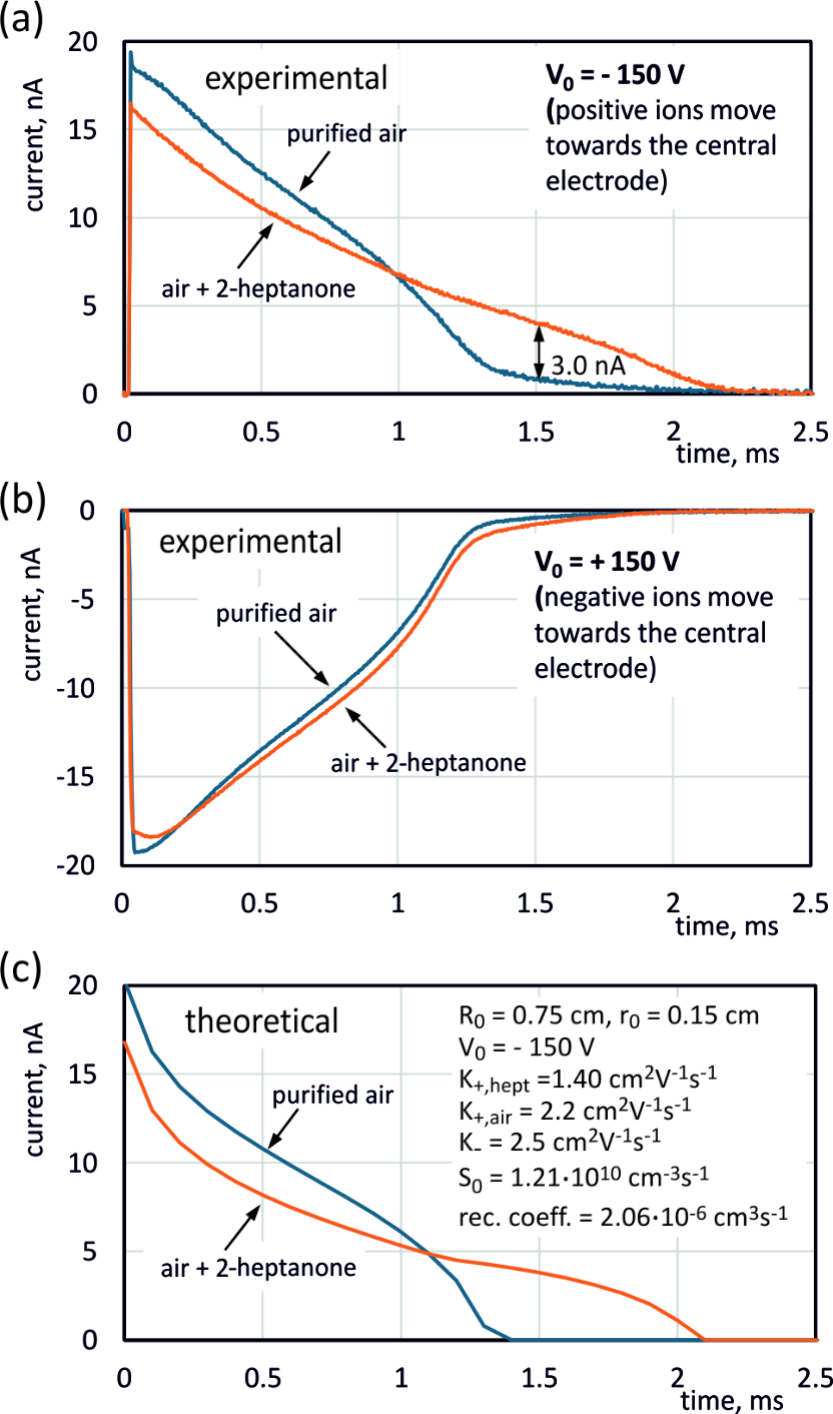
Current pulses measured when the ionization source is periodically switched on for negative (a) and positive (b) voltage applied to the central electrode of the detector. Results of theoretical calculations for negative polarization of the central electrode (c).

The theoretical current waveform after switching on the ion-collecting voltage is shown in Figure 8c. Calculations were performed based on formulas [Disp-formula eq9] and ([Disp-formula eq3]). No ionization during the pulse (*S*(*r*) = 0) and a uniform ion concentration distribution (*n*_0_(*r*) = const) at time t = 0 were assumed.

## Conclusions

The detector we developed is a very simple device designed to detect organic compounds in air that form stable ions with low mobility. Its operation is based on ionization of the sample components and measurement of ionic currents, whose magnitude and time course depend on the ion mobility. The ionization source used in the detector is a miniature X-ray tube emitting radiation with an energy below 5 keV, referred to as soft X-ray. Using such sources does not require special permits or meeting the safety requirements associated with isotopic sources. The ionic currents generated in the detector are stable and relatively high. A useful analytical signal is obtained when the detector is powered by a variable voltage. The detector can operate with the ionization source switched on continuously or with periodic ionization. The latter mode of operation seems particularly advantageous, as it allows for high analytical signal values. Regardless of the detector ’s operating mode, it is possible to determine the type of ions (positive or negative) generated after sample introduction. It is also possible to estimate the mobility of the slowest ions produced in the gas introduced to the detector. The concentrations of the test substances used in the studies were on the order of several dozen ppb. Such concentrations caused a significant change in the analytical signal. It can be expected that the limits of detection for similar substances will be significantly lower. A precise characterization of the detector ’s metrological performance will be possible after conducting more extensive tests, including measurements of calibration curves. Four detector operating modes were analyzed. In each case, the analytical signal was defined as the change in ionic current caused by sample introduction. The analytical signal values for all operating modes are summarized in Table 2.

**Table 2:** Analytical Signal of the Tested Detector for 2-Heptanone at a Concentration of Approximately 29 ppb

Detector operating mode	Analytical signal, nA
static (DC supply)	0.190 ± 0.005
AC voltage supply–instantaneous current	0.70 ± 0.03
AC voltage supply–frequency characteristic	0.34 ± 0.01
Unipolar pulsed voltage + switching of the soft X-ray source	3.00 ± 0.02

The theoretical description of the detector operation is based on solutions of advection equations describing ion transport. Although the model involves substantial simplifications, it reproduces the key detector characteristics, especially the shape and amplitude of the ionic current waveforms. This will allow for future modifications to the detector’s design to optimize its analytical performance.

We believe that the device described in this article could find practical applications. In addition to its role as a simple sensor for detecting hazardous substances in air, the detector could also be used in gas chromatography, similar to an electron capture detector.

## Supplementary Material



## Data Availability

Data will be made available on request.

## Notes

Estimation of the spatial distribution of ionization density, solution of the advection equation, measurement data for methyl salicylate, time courses of the current for higher frequencies, studies of the influence of the supply voltage amplitude on the ionic current course, description of the recombination equilibrium (PDF)
