# Supplementary material for: Mobility-Based Ionization Detector with a Soft X‑ray Source
Source: Anal Chem. 2026 Apr 14;98(16):11955–63. doi: 10.1021/acs.analchem.6c00285 (PMC13298804; doi:10.1021/acs.analchem.6c00285)
Supplement: Supplementary file 1 [file ac6c00285_si_001.pdf]

SUPPORTING INFORMATION

Mobility-based ionization detector with a soft X-ray source

Krzysztof Piwowarski[1], Jarosław Ławreńczyk[1], Michał Kędzierski[2], Jarosław Puton[1,*]

1 - Faculty of Advanced Technologies and Chemistry, Military University of Technology, ul. gen. Sylwestra Kaliskiego 2, 00-908 Warsaw, Poland

2 – Faculty of Civil Engineering and Geodesy, Military University of Technology, ul. gen. Sylwestra Kaliskiego 2, 00-908 Warsaw, Poland

*email: jaroslaw.puton@wat.edu.pl

CONTENTS

Section S1:	Estimation of ionization density distribution	S2
Section S2:	Solving the advection equation - calculating time-dependent ion concentration distributions	S3
Section S3:	Current vs. time dependencies measured for the methyl salicylate	S6
Section S4:	Current vs. time dependencies measured for different amplitudes of supplying voltage	S7
Section S5:	The influence of oscillating ions on the ionic current waveforms	S8
Section S6:	Recombination equilibrium	S9
Section S7:	Signal measured with periodic ionization. Measurement results for methyl salicylate.	S11

Section S1: Estimation of ionization density distribution

The estimation of the ionization density distribution is based on simple geometric considerations. A sketch of the detector geometry is shown in Fig. S1.1a.

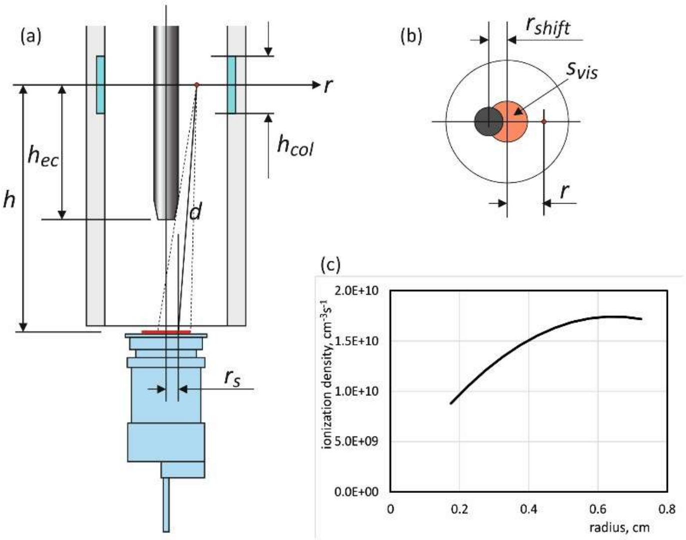

Fig. S1.1. Illustration for estimation of ionization density distribution. Modeling geometry (a), illustration of source obscuration (b) and final modeling result (c).

It is assumed that the X-ray source emits radiation from the surface uniformly. The ionization density at a distance r from the detector axis can calculated as the surface integral over the "visible" source surface s_{vis} (see Fig. S1.1b):

$$S(r) = B \int_{s_{vis}} \cos(\alpha) \frac{1}{d^2} ds = B \int_{s_{vis}} \frac{h}{d^3} ds \tag{S1.1}$$

where B is a constant, α is the angle between the normal to the source surface and the direction of emitted radiation, and d is given by the formula:

$$d^2 = h^2 + (r - r_s)^2$$

For each value of r, the integral appearing in formula (S1.1) was calculated. The integration was performed numerically, two-dimensionally. If the element ds was outside the "visible" region marked in red in Fig. S1.1b, the increment of the integral value was zero. The constant B was determined using the condition that the saturation current (3.01 nA) measured with the ion collector must be equal to the charge of one-sign ions produced in the active volume per unit of time.

$$I_{sat} = 2\pi h_{col} e \int_{r_0}^{R_0} S(r) r dr \tag{S1.2}$$

where h_{col} is the height of the collecting electrode, R_0 and r_0 are the radii of the outer and inner electrodes, and e is the elementary charge. The final result of modeling the ionization density distribution is shown in Fig. S1.1c.

Section S2: Solving the advection equation - calculating time-dependent ion concentration distributions

Calculating the time dependence of the detector current requires finding the time-dependent ion concentration distributions, which are described by the advection equation:

$$\frac{\partial n_i(r,t)}{\partial t} = S(r) - \frac{B_i u(t)}{2r}\frac{\partial n_i(r,t)}{\partial r} \tag{S2.1}$$

where $n_i(r,t)$ is the concentration of i-th type of ions, $S(r)$ is the time-independent ionization density caused by the X-ray source, $u(t)$ is the supply voltage applied to the central electrode (symmetrical square wave with amplitude V_0), and B_i is a factor taking into account the geometry and properties of the ion:

$$B_i = \frac{\pm 2K_i}{\ln(R_0/r_0)} \tag{S2.2}$$

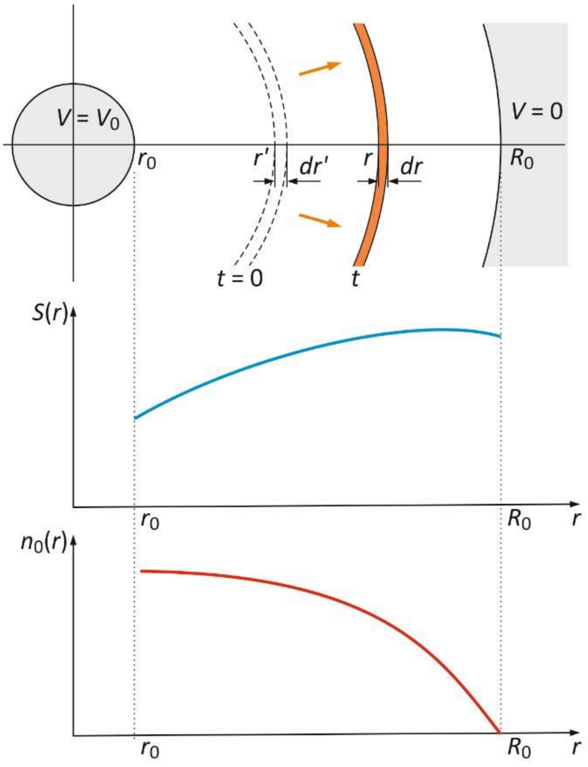

Fig. S2.1. Illustration for deriving the formula describing the time-dependent ion concentration distribution.

The initial condition used in the solution is the stationary concentration distribution $n_{i0}(r)$ occurring before switching the supply voltage. The solution to the problem can be obtained in an analytical form for simple dependencies $S(r)$ and $n_{i0}(r)$. For this purpose, the so-called method of characteristics can

be used, which involves changing variables and reducing the partial differential equation to ordinary differential equations [SI1]. Using the method of characteristics and analytical calculations for the problem described in this article is impossible due to the rather complex shapes of the dependencies $S(r)$ and $n_{i0}(r)$. Numerical solutions of the advection problem based on difference schemes are often problematic due to the instability of the obtained results. Below simple considerations are presented that allow for finding the dependency $n_i(r,t)$ by integrating the source term in the equation (S2.1). The considerations are based on charge (number of ions) conservation.

Let us consider a thin cylindrical layer moving inside the detector with a velocity resulting from its mobility K_i and the electric field strength $E(r)$. For the cylindrical electrode geometry shown in Figure S2.1, the velocity of ions at point r is:

$$\frac{dr}{dt} = E(r)K_i = \frac{1}{2r}B_iV_0$$

(S2.3)

Solving this equation, with the initial condition $r(0) = r'$ allows us to find the position of the ion after time t if it was initially at point r':

$$r(t) = \sqrt{r'^2 + B_iV_0t}$$

(S2.4)

If we neglect the effect of diffusion, the ion concentration in the layer moving between the detector electrodes, as in Figure S2.1, can be described as the sum of two components: (1) the concentration $n_{i,mov}(r,t)$ resulting from the displacement of ions in the electric field from time 0 to t and (2) the concentration $n_{i,src}(r,t)$ associated with the production of new ions during the movement.

Estimation of the first concentration component can be performed if we assume no diffusion. In such case, all ions initially present in the volume $dW' = 2\pi hr'dr'$ will end up in the volume $dW = 2\pi hrdr$ after time t. Differentiating $r(t)$ expressed by formula (S2.4) with respect to r', we obtain:

$$r'dr' = rdr$$

(S2.5)

which means that the volumes dW' and dW are equal and that the concentration of transferred ions is conserved:

$$n_{i,mov}(r,t) = n_{i,mov}(r',0) = n_{i0}(r')$$

(S2.6)

where

$$r'(t) = \sqrt{r^2 - B_iV_0t}$$

(S2.7)

In a cylindrical layer moving as in Figure S2.1, the ion concentration increases due to continuous gas ionization, which corresponds to the $n_{i,src}(r,t)$ component. The increase in concentration over a short time interval dt can be described by the formula:

$$dn_{i,src}(r,t) = S(r)dt$$

(S2.8)

The total increase in ion concentration in the layer can be calculated by integrating the relationship (S2.8) in the range from 0 to t:

$$n_{i,src}(r,t) = \int_0^t S(r')dt \tag{S2.9}$$

Finally, the solution to the advection equation (S2.1) is the expression corresponding to formula (9) in the text of article:

$$n_i(r,t) = n_{i,mov}(r,t) + n_{i,src}(r,t) = n_{i0}\big(r'(t)\big) + \int_0^t S\big(r'(\tau)\big)d\tau \tag{S2.10}$$

The integration with respect to time in equation (S2.10) can be replaced, after changing the variables, by a more convenient integration with respect to the radius:

$$\int_0^t S\big(r'(\tau)\big)d\tau = \frac{2}{B_i}\int_r^{r'} \rho S(\rho)d\rho \tag{S2.11}$$

SI1. Y. Pinchover, J. Rubinstein, An Introduction to Partial Differential Equations. Cambridge University Press (2005). doi:10.1017/cbo9780511801228.

Section S3: Current vs. time dependencies measured for the methyl salicylate

The article includes a graph (Fig. 5) illustrating the effect of the presence of a 2-heptanone sample on the time course of the detector current when the ionization source is continuously switched on. 2-heptanone is a compound with high proton affinity, whose molecules produce only positive ions. Measurements were also conducted for methyl salicylate. In this case, both stable positive and negative ions are generated. The results for methyl salicylate are shown in Figure S3.1. Unlike the results for 2-heptanone, a change in the shape of the ionic current pulse can be seen here for both polarities of the supply voltage.

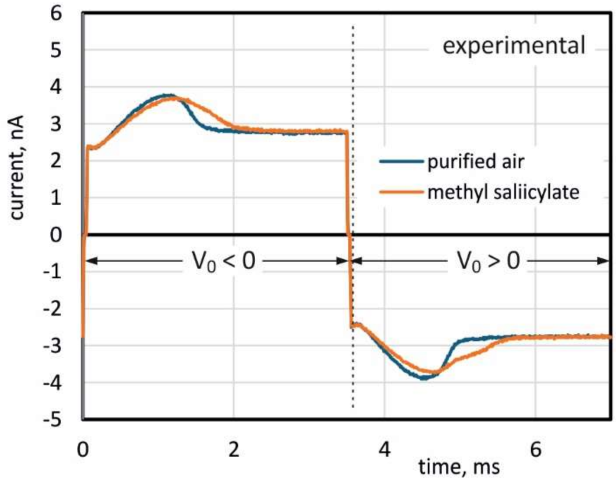

Fig. S3.1. Effect of methyl salicylate addition on the ionic current.

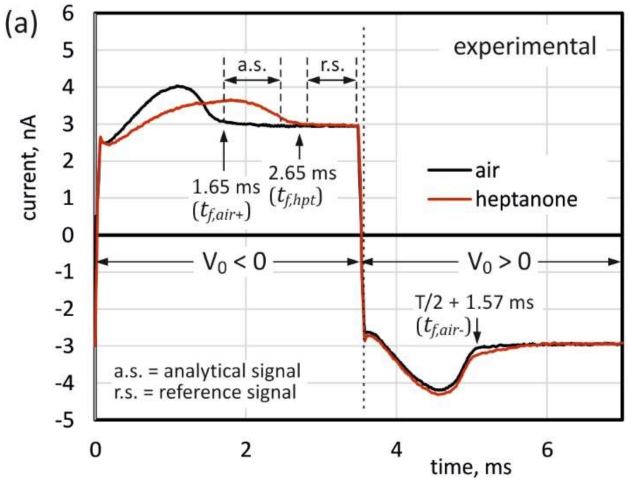

FOR COMPARISON: Figure 5 from main text of the article

Section S4: Current vs. time dependencies measured for different amplitudes of supplying voltage

The shape of the detector current's time course depends not only on the gas composition but also on the supply voltage amplitude. Increasing the amplitude shortens the ion transit time between the electrodes. The effect of voltage amplitude on the detector current's time dependence is shown in Figure S4.1.

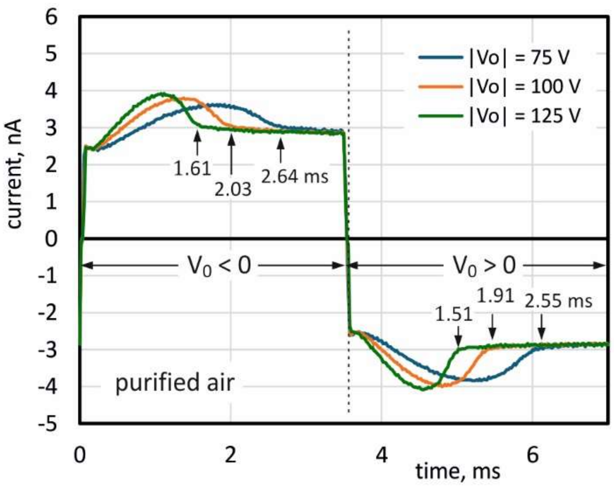

Fig. S4.1. Influence of voltage amplitude on the time dependence of the detector current.

Figure S4.1 shows the durations of current pulses following changes of the detector central electrode's polarity at times = 0 and 3.5 ms. These pulses correspond to the reshaping of ion swarms, and their durations are equal to the ion movement times between the electrodes. Based on these pulses, mobilities for slowest ions can be estimated:

$$K = \frac{(R_0^2 - r_0^2)\ln(R_0/r_0)}{2V_0} t_f \tag{S4.1}$$

Table S4.1 summarizes the mobility calculation results for positive and negative ions. It can be seen that the results obtained for different voltage amplitudes are similar and consistent with the mobility values of positive and negative reactant ions measured using ion mobility spectrometers.

Table S4.1. Estimation of ion mobilities based on times of flight

	positive ions			negative ions		
voltage amplitude, V_0, V	75	100	125	75	100	125
ion's time of flight, t_f, ms	2.64	2.03	1.61	2.55	1.91	1.51
calculated mobility, K, cm^{2}V^{-1}s^{-1}	2.19	2.14	2.16	2.27	2.27	2.38

Section S5: The influence of oscillating ions on the ionic current waveforms

If the detector 's supply voltage frequency is sufficiently high, the ions will not be able to cross the distance between the electrodes during the half a period. A significant change in the shape of the ionic current versus time is then observed (Figure S5.1). An increase in the amplitude of the ionic current is also visible. This increase is caused by the presence of oscillating ions within the detector, which contribute to the induction of current in the electrodes.

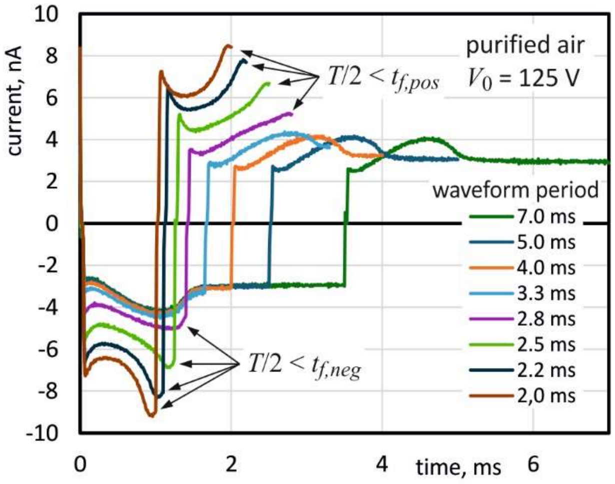

Fig. S5.1. Waveforms of the detector current at higher supply voltage frequencies.

Section S6: Recombination equilibrium

Operating the detector with a periodically switched on and off soft X-ray source allows for the study of the dynamics of achieving so-called recombination equilibrium. In the absence of an electric field and the ionization source turned on, the ion concentration inside the detector increases until the rates of ion production (ionization) and ion decay (recombination) equalize. The dynamics of this process can be described by the simple differential equation with the initial condition:

$$\frac{dn_r}{dt} = S_0 - \alpha n_r^2; \quad n_r(0) = 0 \tag{S6.1}$$

where n_r is the ion concentration (the same for positive and negative ions), S_0 is the ionization intensity, α is the recombination coefficient and t is the time.

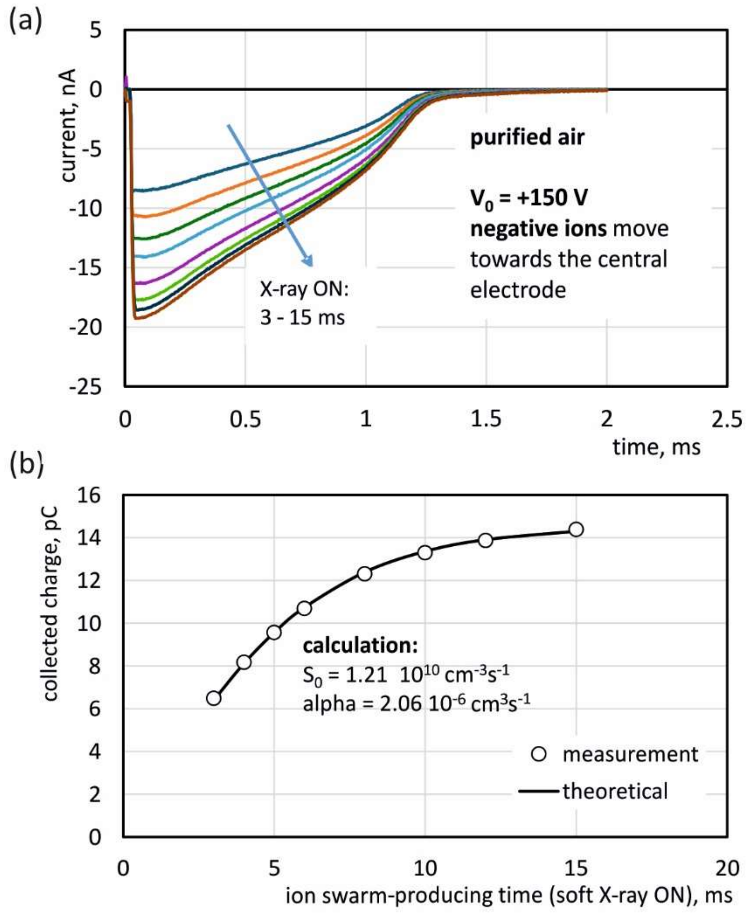

Fig. S6.1. Studies of the achieving of recombination equilibrium. Ion current pulses recorded for different soft X-ray source turn-on times (a) and the dependence of the electric charge corresponding to these pulses on ionization time (b).

The solution to equation (S6.1) is:

$$n_r(t) = \sqrt{\frac{S_0}{\alpha}\frac{1-e^{-t/\tau}}{1+e^{-t/\tau}}}$$

(S6.2)

where

$$\tau = \frac{1}{2\sqrt{S_0\alpha}}$$

The electric charge of ions (of one polarity) Q contained in the active volume of the detector at time t from the moment of switching on the ionization is:

$$Q = \pi(R_0^2 - r_0^2)hn_r(t)e$$

(S6.3)

The sequence of switching on the ionization source and the ion collection voltage was as shown in Figure 7a in the main text of the article. Ion current pulses (Figure S6.1a) obtained for different ionization times were integrated to determine the charge on ions collected from the detector interior. The results of these calculations are shown (as circles) in Figure S6.1b. A theoretical curve, i.e., $Q = f(t_{Xon})$ was fitted to the experimental values using formulas (S6.2) and (S6.3). The values of S_0 and α corresponding to the best fit are given in Figure S6.1b. The entire measurement procedure was carried out for positive voltage polarity on the central electrode, i.e. the opposite of the tests whose results are shown in the article. Our studies show that this change in polarization has practically no effect on the course of the $Q = f(t_{Xon})$ curve and the determined values of S_0 and α.

Section S7: Signal measured with periodic ionization – measurements results for methyl salicylate

The main text of this article includes Figure 8, which shows the effect of 2-heptanone admixture on the shape of current pulses measured for periodically switching on a soft X-ray source. Below (Figure S7.1), a similar relationship is shown for methyl salicylate, a compound that forms stable positive and negative ions. It can be seen that in this case, the shape of the current pulse clearly changes with the polarity of the ion-collecting voltage pulse.

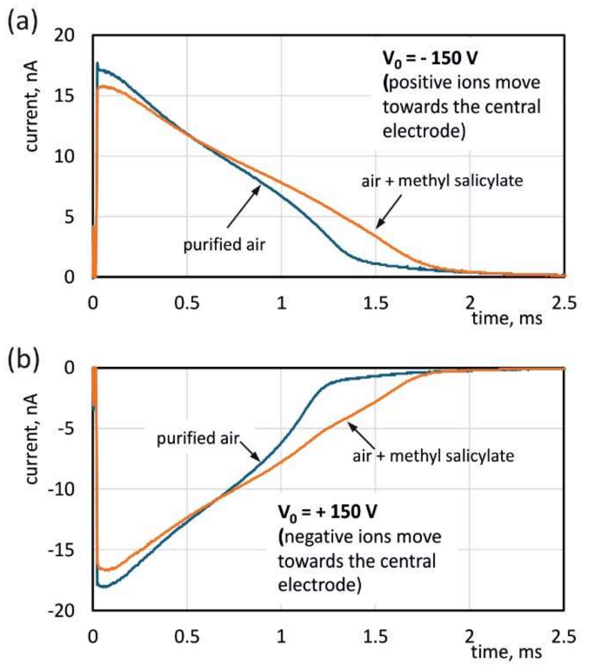

Fig. S7.1. Current pulses measured during periodic switching on of the ionization source for negative (a) and positive (b) voltages applied to the detector 's central electrode. Measurements performed for the methyl salicylate admixture.

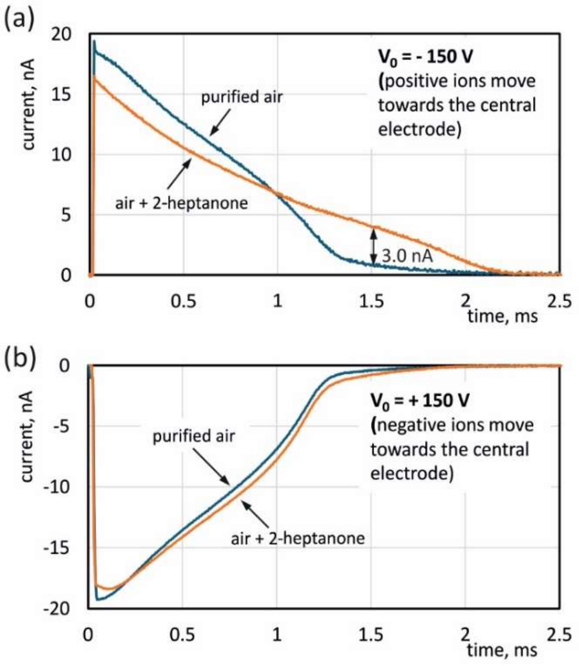

FOR COMPARISON:
Figure 8 from main text of the article
